# DNA methylation alterations in grade II- and anaplastic pleomorphic xanthoastrocytoma

**DOI:** 10.1186/1471-2407-14-213

**Published:** 2014-03-20

**Authors:** Ramón Martínez, F Javier Carmona, Miguel Vizoso, Veit Rohde, Matthias Kirsch, Gabriele Schackert, Santiago Ropero, Werner Paulus, Alonso Barrantes, Antonio Gomez, Manel Esteller

**Affiliations:** 1 Department of Neurosurgery, University of Goettingen, Robert Koch. Str. 40, 37075 Goettingen, Germany; 2 Department of Neurosurgery, University of Dresden, Fetscherstr. 74, 01307 Dresden, Germany; 3 Department of Biochemistry and Molecular Biology, School of Medicine, University of Alcalá, Carretera Madrid-Barcelona Km. 33.6, 28871 Madrid, Spain; 4 Institute of Neuropathology, University Hospital Muenster, Domagkstr. 17, 48149 Muenster, Germany; 5 Institute of Neuropathology, University of Goettingen, Robert Koch. Str. 40, 37075 Goettingen, Germany; 6 Cancer Epigenetics and Biology Program (PEBC), Bellvitge Biomedical Research Institute (IDIBELL), Hospital Duran i Reynals, Av. Gran Via de L’Hospitalet 199-203, 08907 Barcelona, Catalonia, Spain; 7 Department of Physiological Sciences II, School of Medicine, University of Barcelona, 08907 Barcelona, Catalonia, Spain; 8 Institució Catalana de Recerca i Estudis Avançats (ICREA), Barcelona, Catalonia, Spain

**Keywords:** Epigenetics, DNA methylation, Glioblastoma, Pleomorphic xanthoastrocyma

## Abstract

**Background:**

Pleomorphic xanthoastrocytoma (PXA) is a rare WHO grade II tumor accounting for less than 1% of all astrocytomas. Malignant transformation into PXA with anaplastic features, is unusual and correlates with poorer outcome of the patients.

**Methods:**

Using a DNA methylation custom array, we have quantified the DNA methylation level on the promoter sequence of 807 cancer-related genes of WHO grade II (n = 11) and III PXA (n = 2) and compared to normal brain tissue (n = 10) and glioblastoma (n = 87) samples. DNA methylation levels were further confirmed on independent samples by pyrosequencing of the promoter sequences.

**Results:**

Increasing DNA promoter hypermethylation events were observed in anaplastic PXA as compared with grade II samples. We further validated differential hypermethylation of *CD81, HCK, HOXA5, ASCL2* and *TES* on anaplastic PXA and grade II tumors. Moreover, these epigenetic alterations overlap those described in glioblastoma patients, suggesting common mechanisms of tumorigenesis.

**Conclusions:**

Even taking into consideration the small size of our patient populations, our data strongly suggest that epigenome-wide profiling of PXA is a valuable tool to identify methylated genes, which may play a role in the malignant progression of PXA. These methylation alterations may provide useful biomarkers for decision-making in those patients with low-grade PXA displaying a high risk of malignant transformation.

## Background

Pleomorphic xanthoastrocytoma (PXA) is a rare WHO grade II tumor accounting for less than 1% of all astrocytomas. They are usually hemispheric, and often they affect children and young adults (median age 26 years) with a frequent history of chronic epilepsy at presentation [1-3]. The majority of the tumors occur in the supratentorial compartment, mostly in the temporal lobe [1,4]; rarely, they were observed in thalamus, cerebellum, sellar region and spinal cord [1,5-7].

Histologically, PXA shows a pleomorphic appearance, an intense reticulin network and lipid deposits within ovoid and spindled tumor cells. Giant tumor cells, eosinophilic granular bodies and lymphocytic infiltrates are also observed. Immunopositivity for glial fibrillary acidic protein (GFAP) is virtually always encountered. The mitotic activity is absent or very low and MIB-1 labeling index is frequently <1%.

The biological behavior is usually benign with a 10-years survival rate of 70% and a recurrence-free lapse of 61% [8]. Nevertheless, gradeII PXA may undergo malignant transformation in up to 15-20% [8]. Thus, PXA with elevated mitotic activity (≥ 5 mitoses per 10 high-power fields) and/ or presence of necrosis has been classified as “PXA with anaplastic features” [1,8]. In these cases, the rate of recurrence has been observed to be much higher and the survival time clearly shorter [8]. These malignant forms are largely responsible for the mortality rate from the disease at 10 years. This aspect is particularly relevant considering that PXA is often encountered in children and young adults.

Epigenetic alterations are able to modulate gene activity without affecting their nucleotide sequence. Aberrant methylation has been recognized as a hallmark of human cancer, and DNA methylation patterns are altered in all types of cancers analyzed, affecting virtually all cellular pathways trough methylation-mediated silencing of regulator genes such as *VHL, p16*^*INK4a*^*, E-cadherin, hMLH1, BRCA1* and *LKB1*, and many others [9,10]. In cancer cells a wide-ranging process leading to global changes in DNA methylation patterns takes place. Specifically, a global hypomethylation process happening mainly at intergenic regions and repetitive sequences, as well as local promoter DNA hypermethylation that affects usually unmethylated CpG-rich DNA sequences mapping to tumor-suppressor genes. Thus, their activity is abrogated by means of transcriptional repression [11,12].

Although an increasing interest about aberrant DNA methylation in gliomas exists, the epigenetic profile of astrocytic tumors remains only partially devised, which is especially true for PXA. Widespread hypomethylation [13] might play a role in the pathogenesis of gliomas through activation of oncogenes, loss of imprinting, or the promotion of genomic instability which, in turn exacerbates the tumorigenic phenotype of the cell. Moreover, the importance of aberrant DNA methylation of CpG island promoter regions in the pathogenesis of gliomas, oligodendrogliomas, ependymomas and pituitary adenomas is highlighted by the observation of hypermethylation of a wide variety of genes associated with tumor suppression (*RB1, VHL, EMP3, RASSF1A, CITED4, BLU*), cell cycle regulation (*p16*^*INK4a*^*, p15*^*INK4b*^), DNA repair (*MGMT, hMLH1*), and tumor invasion and apoptosis (*DAPK, TIMP3, CDH1, SOCS3*) [13-24]. *MGMT* is another good example of a DNA repair gene undergoing methylation-mediated inactivation in human cancer [25], including GBM [26].

It has recently become evident that the methylation signature of astrocytic tumors appears to be class-specific. Analyzing a panel of 7 genes (*CDKN2B, PTGS2, CALCA, MYOD1, THBS1, TIMP3* and *CDH1*) Uhlman and colleagues observed differences in the methylation status in astrocytomas WHO grades II, III and IV [27]. Concerning PXA there is almost no data available from the literature reporting on epigenetic signatures and only a specific report focusing on *MGMT*[28] methylation status has been published so far.

In the present study we have analyzed the DNA methylation profiles of PXA WHO grade II (n = 9) and PXA with anaplastic features (n = 2). Brain tissue obtained by epilepsy neurosurgical procedures in patients without brain tumors (n = 10) and GBM consecutive samples (n = 87, previously analyzed [29]) were included in the DNA methylation analysis. For this purpose, we have investigated the DNA methylation alterations following a microarray-based DNA methylation approach as well as pyrosequencing of selected genes for further validation.

## Methods

### Patient samples and controls

The study was approved by the Ethics Committee of the School of Medicine, University of Göttingen (project number 11/8/13), and patients provided written informed consent to participate on the study, as well as for publishing the images and clinical information. All PXA (n = 13 tumors, 12 patients) and GBM patients (n = 87) had undergone surgery with the goal of maximal possible tumor resection. One anaplastic PXA (PXA-5) was the local relapse of a grade II PXA (PXA-4), one year after complete tumor resection (Table 1). All patient IDs have been appropriately codified to ensure privacy protection. Normal human adult brain (NB, n = 10) tissue obtained from epilepsy neurosurgical procedures, and post-mortem from healthy individuals was included in the study as normal control. Tumor samples were frozen in liquid nitrogen and stored at –80°C. Tumor tissue was evaluated by experienced neuropathologists according to the 2007 WHO classification criteria. DNA from tumor specimens was isolated applying the QIAamp® DNA Mini Kit (Qiagen, Hilden, Germany). Informed consent for samples and data analysis was obtained from each patient or the patient’s carer. The design of this analysis conforms to standards currently applied in Germany. Survival times were collected for all cases and were calculated from the time of diagnosis to death, or last contact in the case of living patients.

**Table 1:** Histopathological characterization of PXA patients

**PXA patients**	**Diagnosis**	**Gender, age**	**ICH features**	**Brain localization**
			BRAF V600E negative (no mutation)	
			CD34 positive.	
PXA-1	PXA WHO grade II	18y., female	GFAP positive.	right frontal
			MIB-1:1%	
			IDH1 R132H negative (no mutation).	
			GFAP positive.	
PXA-2	PXA WHO grade II	17y., female	MIB-1: 1%.	right temporal
			No tumor tissue available for BRAF/CD34 characterization	
			BRAF V600E positive (mutated)	
			CD34 focal positive	
PXA-3	PXA WHO grade II	34y., female	GFAP. Positive.	left temporal
			MIB-1: <1%.	
			IDH1 R132H negative (no mutation)	
			BRAF V600E negative (no mutation)	
			CD34 positive	
			Mitotic index <5 mitoses/10 HPF	
PXA-4 precursor of PXA-5	PXA WHO grade II	59y., male	Immunopositivity for GFAP, S100, MAP2,	right parietal
			vimentin and EMA	
			MIB-1: 5-10%	
			IDH1 R132H negative (no mutation)	
			BRAF V600E negative (no mutation)	
			CD34 positive	
			Mitotic index >5mitoses/10 HPF	
PXA-5	PXA with anaplastic features	59y., male	Immunopositivity for GFAP, S100, MAP2,	right parietal
			vimentin and EMA	
			MIB-1: 10-15%	
			IDH1 R132H negative (no mutation)	
			BRAF V600 E negative (no mutation)	
			CD34 positive	
PXA-6	PXA with anaplastic features	22y., male	GFAP positive	right temporal
			MIB-1: 5%.	
			IDH1 R132H negative (no mutation)	
			BRAF V600 E negative (no mutation)	
			CD34 positive	
PXA-7	PXA WHO grade II	44y., female	Immunopositivity for GFAP	right temporal
			MIB-1: 1%.	
			IDH1 R132H negative (no mutation)	
			BRAF V600 E negative (no mutation)	
			CD34 positive	
PXA-8	PXA WHO grade II	20y., male	Immunopositivity for GFAP.	right parieto-occipital
			MIB-1: <1%.	
			IDH1 R132H negative (no mutation)	
			BRAF V600 E negative (no mutation)	
			CD34 positive	
PXA-9	PXA WHO grade II	78y., female	Immunopositivity for GFAP.	right temporo-parietal
			MIB-1: <1%.	
			IDH1 R132H negative (no mutation)	
PXA-10	PXA WHO grade II	15y., female	BRAFV600E negative (no mutation)	right occipital
			CD34 positive	
			Immunopositivity for GFAP.	
			MIB-1: 1%.	
PXA-11	PXA WHO grade II	52y., male	BRAFV600E negative (no mutation)	left occipital
			CD34 positive	
			Immunopositivity for GFAP.	
			MIB-1: < 1%.	
PXA-12	PXA WHO grade II	17 y., male	BRAFV600E positive (mutated)	left parietal
			CD34 negative	
			Immunopositivity for GFAP.	
			MIB-1: 1%.	
PXA-13	PXA WHO grade II	37 y., female	BRAFV600E positive (mutated)	right parietal
			CD34 focal positive	
			Immunopositivity for GFAP	
			MIB-1: < 1%	

Patients undergoing surgical resection of diagnosed pleomorphic xanthoastrocytoma of diverse grade were investigated for specific features characteristic of this tumor type.

### DNA methylation profiling using universal BeadArrays

DNA methylation profiling was performed with the GoldenGate Methylation Cancer Panel I assay (Illumina Inc., San Diego CA) on a total of 8 WHO grade II PXA, 2 anaplastic PXA, 87 GBM samples and 10 normal brain tissue samples as control. The panel was developed to assay 1,505 CpG sites selected from 807 cancer-related genes, including oncogenes and tumor suppressor genes, imprinted genes, genes involved in various signaling pathways, and those responsible for DNA repair, cell cycle control, metastasis, cell migration and invasion, differentiation and apoptosis.

Methylation assay was performed following manufacturer’s instructions. Briefly, bisulphite conversion of DNA samples was carried out using the EZ DNA methylation kit (Zymo Research, Orange, CA). After bisulphite treatment, the remaining assay steps were performed using Illumina-supplied reagents and conditions. We excluded 84 CpGs mapping to X chromosome to avoid gender-specific bias. Additionally, we evaluated the detection probabilities (comparing signal intensities against background noise) for all CpGs and excluded those probes with values of *P* > 0.01 in more than 10% of cases. In the final analysis, 1,390 CpGs mapping to 762 genes were used in the subsequent statistical analyses.

### Data analysis and definition of DNA methylation patterns

For further analyses, only the 1,390 autosomal CpGs that met our quality criteria were used. In order to distinguish groups of patients according to their DNA methylation patterns, we selected only the informative probes, that is, considering those probes with SD > 0.05 between patients. We sought to identify genes affected by DNA hypermethylation in the precursor grade II PXA (PXA-4) that are further maintained in the anaplastic relapsed tumor (PXA-5) and in the analyzed glioblastoma samples, in comparison with the average values of grade II PXA cases and normal brain. The glioblastoma patients used in this study for comparison purposes were described in detail elsewhere [29]. To this aim, we set up a threshold increment of 30% in methylation, as has been previously reported to result in expression differences [30], when comparing the averaged anaplastic and precursor PXA samples (PXA-4 and PXA-5), the averaged grade II PXA cases (PXA1-3) and the GBM patients. Candidates that exhibit (i) an unmethylated status in normal brain (β ≤ 0.2, SD < 0.1), (ii) a difference in DNA methylation (DM) between averaged grade II and anaplastic cases higher than DM > 30%, and (iii) are also methylated in the averaged GBM group (β > 0.4, SD < 0.1), were selected for validation. Furthermore, we investigated DNA hypermethylation events specific for precursor (PXA-4) and anaplastic (PXA-5) PXA cases that retained normal levels in the rest of the samples, including GBM, and therefore represent specific DNA hypermethylation events of this tumor entity.

### Pyrosequencing and bisulfite genomic sequencing

In order to validate the results obtained from the DNA methylation array, pyrosequencing was performed on selected candidate genes as has been previously described, on grade II PXA samples, anaplastic PXA samples (PXA 4-6), as well as in glioblastoma and normal brain samples*.*

Genomic DNA was converted using the EZ DNA Methylation Gold kit (Zymo Research, Orange, CA, USA). DNA methylation in clinical samples was studied by pyrosequencing, which was performed on bisulphite-treated DNA extracted from formalin-fixed paraffin-embedded (FFPE) samples. Specific primers were designed using the PyroMark Assay Design Software (QIAGEN-version 2.0.01.15) for to examine the methylation status of particular CG sites covering the candidate genes promoter regions (Additional file 1: Table S1). Pyrosequencing primer sequences were designed to hybridize with CpG-free sites to ensure methylation-independent amplification. PCR was performed with primers biotinylated to convert the PCR product to single-stranded DNA templates. We used the Vacuum Prep Tool (Biotage) to prepare single-stranded PCR products according to the manufacturer’s instructions. Pyrosequencing reactions and quantification of DNA methylation were performed in a PyroMark Q96 System version 2.0.6 (QIAGEN) including appropriate controls. For bisulfite genomic sequencing of *MGMT* promoter sequence, specific sets of primers were designed using the Methyl Primer Express software (Applied Biosystems) (Fwd: GGTAAATTAAGGTATAGAGTTTTAGG; Rev: ACCCAAACACTCACCAAAT), and a minimum of eight clones were sequenced. It allows a positive display of 5- methyl cytosines in the gene promoter after bisulfite modification as unmethylated cytosines appear as thymines, while 5-methylcytosines appear as cytosines in the final sequence.

### Statistical analysis and Gene ontology analysis of differentially methylated genes

In order to define DNA methylation patterns between and inside groups of samples, statistical comparisons were performed. Mann-Whitney U-test (False Discovery Rate, FDR < 0.05) and Fisher’s exact test were performed to compare differences between groups of glioblastoma and normal brain sample sets, depending on the data types of the variables being examined. DNA methylation values of glioblastoma and normal brain samples were averaged for comparative purposes. Furthermore, the genes found differentially methylated on anaplastic PXA cases exhibited a SD < 0.1 in the GBM cohort, and were all detected as significantly hypermethylated in a large series study [29]. Due to sample size, average values and standard deviation of PXA were compared to the values of DNA methylation patterns of glioblastoma and normal brain samples sets. Analyses were performed with SPSS (version 11.5, SPSS Inc., Chicago, IL., USA). GO enrichments for biological process ontology were calculated using the *GOStats* package under R statistical software. Those terms below an adjusted (Benjamini-Hochberg correction) p-value below 0.01 were selected and considered significant.

## Results

### Clinical, histological and genetic characterization of patient samples

**Figure 1:**
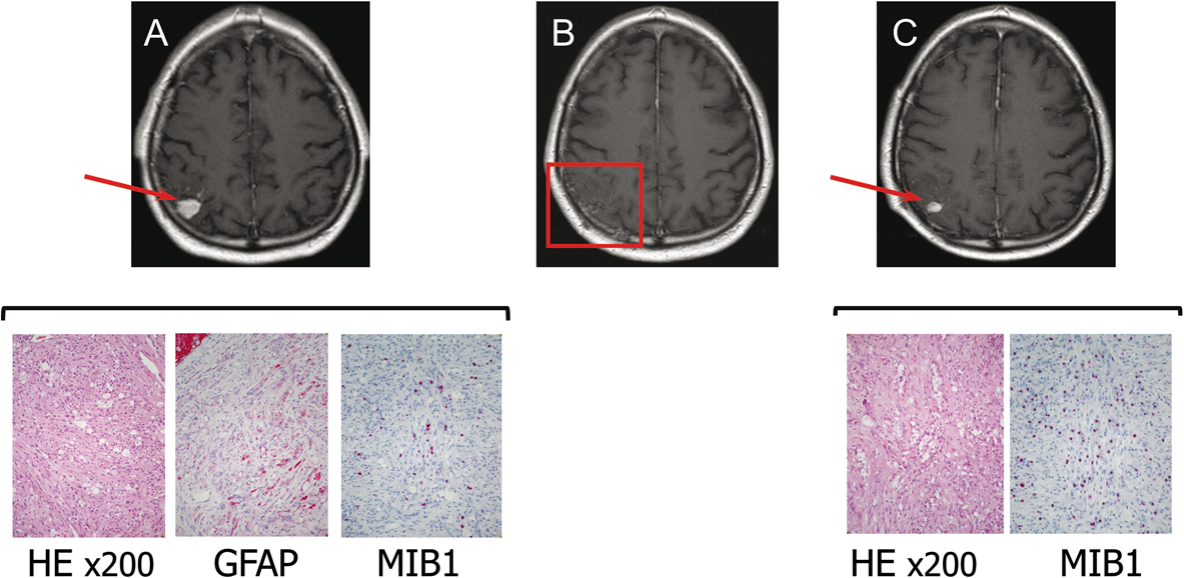
**Immunohistochemical characterization of grade II PXA and associated anaplastic PXA.** Upper row: T1-weighted, gadolinium-enhanced axial MRI showing the right parietal PXA at presentation **(A)**, after surgical resection **(B)** and at the time of local relapse **(C)**. Lower row: Photomicrographs showing histological and immunohistochemical features of the grade II PXA and grade III PXA with anaplastic features (HE, GFAP and MIB-1. The last one showed a higher positivity of 20% in the anaplastic PXA, whereas it was 10% in the grade II PXA).

In PXA patients, the male: female ratio was 1:0.8, in GBM patients was 1:0.7, and in control cases 1:1.3. The median age at diagnosis was in PXA patients 26 years (Table 1), in GBM patients 60.6 years, and in control cases 52.1 years. All PXA patients presented with a 4-8-week history of epileptic seizures, dizziness and headache, after that MRI diagnosis (Figure 1) was performed and the tumors were diagnosed. All PXA patients underwent complete surgical resection. The one patient diagnosed with a PXA with anaplastic features (PXA-5) was the local relapse of one of the grade II PXA (PXA-4), one year after complete tumor resection. Albeit being an outlier for the median age at diagnosis, cases of PXA diagnosed in adults and elderly patients have been documented previously as well [31,32]. After resection of the anaplastic PXA, adjuvant fractionated radiotherapy (59Gy) and chemotherapy with temozolomide were performed. All analyzed PXA show the typical characteristic of this type of lesion (Table 1). Figure 1 shows comparative immunohistochemical investigations with hematoxylin & eosin (HE), glial fibrillary acidic protein (GFAP) and MIB-1 in the cases of grade II PXA and associated anaplastic PXA, and illustrative examples of histological assessment of BRAF V600E mutation (PXA-3) and CD34 immunoreactivity (PXA-1) are included on Additional file 2: Figure S1. Patients with glioblastoma had undergone standard therapy with gross-total surgical resection followed by adjuvant fractionated radiotherapy (median 59 Gy) and chemotherapy with temozolomide (Stupp regime).

### Detection of candidate-genes differentially methylated in malignant PXA

**Figure 2:**
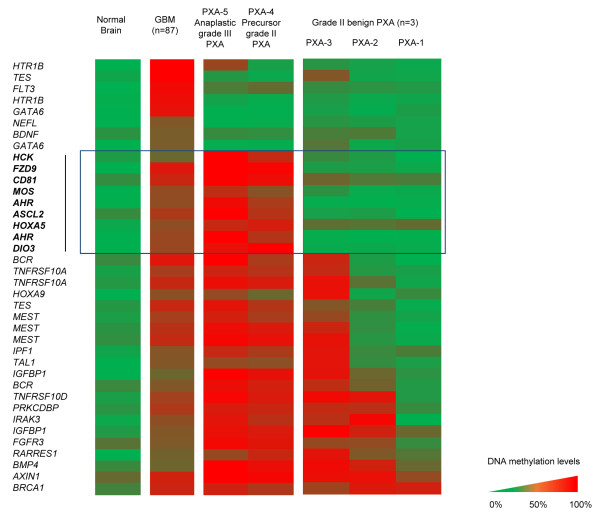
**DNA methylation alterations on grade II and malignant PXA samples.** Based on methylation frequencies, tumor samples can be categorized on three groups: (1) grade II PXA (PXA 1-3); (2) precursor and anaplastic PXA (PXA 4-5) and (3) GBM. PXA-4 and PXA-5 samples show increased DNA methylation when compared with grade II PXA. A set of genes was observed to be commonly hypermethylated in anaplastic PXA, its grade II PXA precursor and GBM, whereas being unmethylated in all other grade II PXA and normal brain (squared in blue). Color scale shows methylated (red) and unmethylated (green) status of the probes.

Aiming to recognize changes attributable to malignant transformation of PXA into GBM, we sought to identify specific changes between grade II and anaplastic PXA cases. To this end, we explored the DNA methylation profiles in PXA patients, restricting the analysis to genes being unmethylated (β < 0.2, SD < 0.1) in NB and methylated in GBM (β > 0.4, SD < 0.1) in any of the probes. As a result, a pattern of progressive hypermethylation was recognized, allowing the categorization of the analyzed cases in three groups: (1) grade II PXA samples without further recurrence; (2) anaplastic PXA cases and the corresponding grade II PXA precursor tumor; and (3) GBM (Figure 2). PXA samples showed a progressive increase in the frequency of hypermethylated CpGs correlating with the presence of malignant features (>5 mitoses pro high-power field and/or necrosis). A considerable number of hypermethylated genes stand out the anaplastic PXA cases and the one grade II PXA precursor tumor (PXA-4), whereas no hypomethylation events were detected. Interestingly, we found a series of genes showing DNA hypermethylation (DM > 30%) at gene promoters restricted to the malignant PXAs (Figure 2) as compared with grade II samples. Specifically, this set of genes (*CD81, HCK, HOXA5, ASCL2, TES, AHR, DIO3, FZD9* and *MOS*) exhibited large DNA methylation increments when comparing grade II PXAs and grade II PXA-4 case precursor of the anaplastic PXA-5; were consistently methylated in anaplastic PXA and in GBM patients as well (β > 0.4) but unmethylated (β < 0.2) in normal brain, thus indicating an association between increasing rate of hypermethylation events and the presence of histological malignant features.

### Pyrosequencing validation of the investigated genes

**Figure 3:**
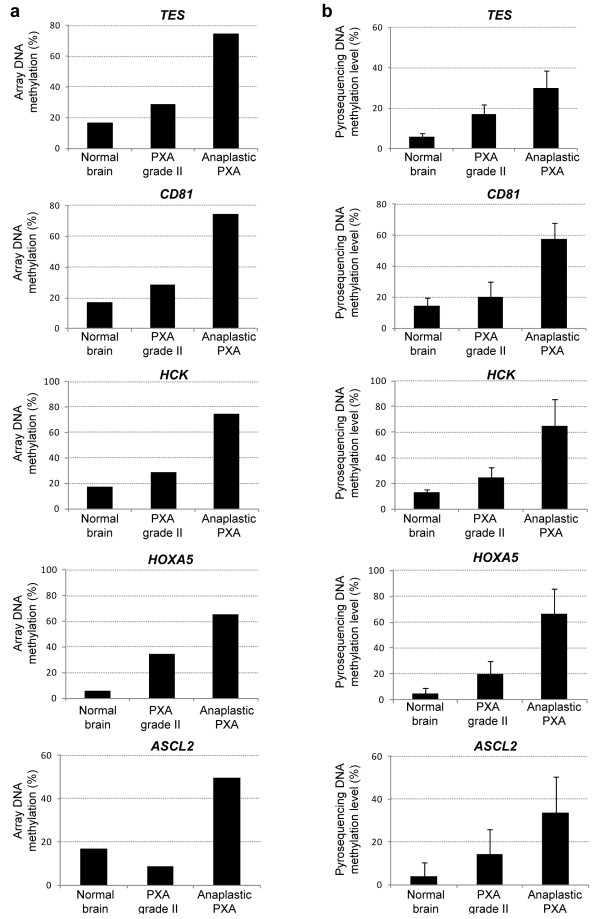
**Validation of DNA hypermethylation events in malignant transformation of PXA.** Representation of DNA methylation levels exhiited by the five selected markers on the GoldenGate DNA methylation assay **(a)** and validated by pyrosequencing on an independent set of samples **(b)**.

DNA methylation values obtained from the GoldenGate methylation assay were validated by pyrosequencing in those samples used for the discovery phase (PXA 4, 5), as well as on an independent set of validation samples (PXA 6-11). Specifically, we carried out validation of the candidate genes differentiating grade II PXA cases and anaplastic PXAs (including the one corresponding precursor grade II PXA), which were hypermethylated in GBM cases as well. For technical limitations, from the nine genes identified, pyrosequencing could be performed for five of them (*CD81, TES, HOXA5, ASCL2, HCK*). The results obtained on the GoldenGate assay (Figure 3A) were highly consistent with those obtained by pyrosequencing on the discovery and validation samples (Figure 3B). The five genes exhibited comparable DNA hypermethylation gains in the anaplastic PXA cases and were unmethylated in normal brain and grade II PXA cases (Figure 3B). In addition, analysis of *MGMT* promoter hypermethylation was also performed, and comparable promoter DNA hypermethylation was found in the anaplastic PXA samples as well as in the one grade II tumor further relapsing as an anaplastic PXA (Additional file 3: Figure S2).

### Gene ontology (GO) analysis of genes differentially methylated in malignant PXA

**Figure 4:**
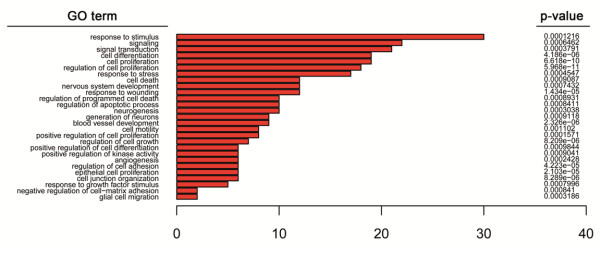
**Gene ontology analysis of the genes hypermethylated in anaplastic PXA.** When comparing the precursor grade II (PXA-4) and anaplastic grade III (PXA-5) PXA tumors with average methylation observed in normal brain, specific gains of methylation involve specific nervous system pathways, as well as other related with oncogenic potential such as cell proliferation, motility and differentiation. Scale bar at the bottom indicates number of genes involved on each biological process. Annotated biological processes were selected among the statistically significant GO-terms resulting from the analysis (BH-adjusted p value < 0.01).

In order to gain insights into the impact of DNA hypermethylation on each of the established categories, we extracted the specific DNA methylation increases for each sample set, taking the DNA methylation profile of normal brain (NB) as a reference. By comparing normal brain and malignant PXA cases, we identified 140 probes mapping to 116 unique genes gaining methylation (β > 30%; NB < 20%) in the malignant PXA and precursor lesion (Additional file 4: Table S2), and 49 probes (42 unique genes) showed hypermethylation specifically in the anaplastic PXA cases. These genes retained an unmethylated status in GBM and grade II PXA, indicating that these DNA hypermethylation changes are inherent to these tumor samples. As genes studied with the methylation-specific BeadArray were selected for their involvement in cancer, by definition they will be enriched for functions deregulated in cancer. Even taking this limitation into consideration, gene ontology analyses of the changes identified between malignant PXA and normal brain, were observed to be preferentially affecting genes involved in neuronal regulation, including response to stimulus or protein phosphorilation; in addition to pathways related with oncogenic progression, including cell motility and cell adhesion -cadherin and integrin signaling pathways-, cell proliferation -Wnt signaling components-, and angiogenesis among the affected signaling circuits (Figure 4). These pathways were considerably overlapping with those affected by DNA hypermethylation in GBM patients, as has been previously described [29]. When focusing on changes affecting grade II PXA, only 23 probes mapping to 19 unique genes where found differentially methylated in comparison with NB (Additional file 5: Table S3) and no enrichment in biological functions resulted, confirming their benign nature.

## Discussion

In the present study, we sought to explore the epigenetic alterations associated to grade II PXA and those occurring associated with the acquisition of histological malignant features, such as mitoses (as above mentioned) and/or necrosis. PXA WHO grade II is a slow-growing astrocytic tumor, which is considered benign and presents a 10-years survival rate of 70% and a recurrence-free lapse of 61% [8,33]. However, up to 20% PXA will develop anaplastic features and may further progress to secondary glioblastoma, exhibiting much more aggressive phenotype and dropping significantly survival rates with a median survival time of 15 months [8].

DNA methylation changes, and moreover those associated to malignant transformation of grade II PXA, had not been investigated previously. In the present study, we analyzed DNA hypermethylation on the promoter sequences of a panel of cancer-related genes in order to investigate epigenetic alterations associated to this process. To our knowledge, only MGMT methylation status has been previously studied in PXA [28], while the data on the epigenetic regulation of *CD81*, *HCK*, *TES*, *HOXA5* and *ASCL2* in PXA patient have not been documented before. Despite the low cohort size, we observed comparable increases on the DNA methylation levels in independent samples used for validation. Interestingly, when analyzing differences in DNA methylation affecting each sample type, we found a much higher number of changes occurring in anaplastic PXA and the grade II precursor tumor (116 genes) as compared with fewer occurring in all other grade II PXA (19 genes) samples (Additional file 4: Table S2 and Additional file 5: Table S3). Accumulation of genetic alterations has been found associated to the pathogenesis and progression of astrocytic tumours [34], and concomitantly, accumulation of epigenetic lesions is present as well. Among the changes identified, we observed a set of DNA hypermethylation events in anaplastic PXA, its corresponding precursor grade II tumor, overlapping with DNA methylation alterations also found in GBM (Figure 3). Several studies have previously reported frequent epigenetic disruption of *CD81* in glioblastoma [29,35], supporting its tumor-suppressor roles in this cancer type. Moreover, promoter hypermethylation of the gene coding for Testin (*TES*) was also reported by us [29] and others [36,37]. Its role acting as a negative regulator of cell growth supports a role for tumor-suppression, as has been proposed in diverse cancer types including ovarian cancer [38] and acute lymphocytic leukemia [39]. DNA hypermethylation of the transcription factor *ASCL2* has been identified in other cancers [40] and is involved in the regulation of gene expression in the central and peripheral nervous system [41]. Additionally, the pathways deregulated by DNA methylation changes (Figure 4) showed great consistency with those targeted in GBM, and are concordant with previous observations reported by us and others [29,37]. This data suggest that malignant progression of grade II PXA towards anaplastic relapses could be triggered by molecular mechanisms also involved in GBM. This change of methylation status during malignant progression could be also confirmed at the end-point of anaplastic PXA cases (Additional file 4: Table S2). The subset of genes validated in our study (*CD81, TES, HOXA5, ASCL2,* and *HCK*) were not affected by DNA hypermethylation in the grade II patients analyzed, and therefore could be specifically associated to the progression of the disease in this patient (Figure 2 and 3). On the other hand, we cannot rule out that the epigenetic alterations observed could be attributable to individual-specific DNA methylation phenomena; however, methylation of these genes has also been found in GBM in larger population studies [29,35-37,42], thus indicating that they are most probably involved in the pathogenesis and malignant progression of astrocytic tumors.

Of note, considerable promoter hypermethylation was found in *MGMT* promoter region, both in the precursor and the anaplastic tumors (Additional file 3: Figure S2). A recent report analyzing the methylation status of *MGMT* in 11 grade II PXA concluded that this event was infrequent in PXA [28]. Albeit this finding needs to be assessed in larger population studies, the extensive promoter hypermethylation we found in the anaplastic PXA patient supported the indication of chemotherapy with temozolomide in a similar pattern as it is in other malignant tumors of astrocytic lineage [40].

The pathogenesis of PXA is largely unknown. Nevertheless, a series of molecular studies have described genomic alterations in PXA patients [28,42-48] Chromosomal gains and losses have been associated with PXA pathogenesis, although prevalent losses -frequently involving chromosomes 7 and 9- were observed in grade II astrocytomas of poor prognosis [28,42-44], accounting for a potential inactivating mechanism of tumor suppressor genes. Further genetic studies have also unveiled the high frequency of BRAF V600E mutations in WHO grade II PXAs and PXAs with anaplastic features (65 and 66% of cases, respectively) [48], as well as homozygous deletion of CDKN2A/p14(ARF)/CDKN2B in six out of ten tumors analyzed in a different cohort [46]. DNA methylation alterations have been scarcely analyzed in PXA. Marucci and colleagues examined *MGMT* promoter methylation in 11 grade II PXA [28], but, to our knowledge, no additional studies have been done to examine epigenetic alterations in this setting. Methylation markers in a variety of human cancers have proved trustworthy in clinical trials for diagnostic and prognostic purposes. In other solid tumors, DNA methylation markers have shown their relevance in early diagnosis, prognosis of tumor progression, or response to therapy and chemo-resistance [49,50]. For instance, DNA methylation profiles were shown to correlate with clinical parameters; specifically hypermethylation of *GATA6* transcription factor was found associated with poor survival in GBM patients [35], or hypermethylation of the pro-apoptotic CASP8 is a differential feature of GBM relapses [51]. Though limited by the size of the populations analyzed, our study suggests that DNA hypermethylation mediated silencing of tumor suppressor genes in PXA could be a relevant event contributing to malignant progression, as also defined by other diffuse astrocytic tumors. The diagnosis of grade II PXA at a high risk to recur as a malignant tumor widens the therapeutic window for intervention, in the form of early onset of adjuvant chemotherapy, even at a grade II tumor stage. Thus, in order to depict biomarkers with prognostic value on PXA patients, broader studies should be undertaken in this and further low-grade astrocytic tumors with risk to undergo malignant transformation.

## Conclusions

In the present study, though limited by sample constraints, we have identified promoter hypermethylation of *CD81, HCK, HOXA5, ASCL2* and *TES* genes in anaplastic cases compared to grade II PXA. These events could potentially contribute to malignant progression of PXA, since similar methylation increases were not observed in grade II cases. Moreover, hypermethylation of these genes were observed in GBM as well, suggesting widespread epigenetic mechanisms of malignancy. This study should be further confirmed in larger population series aiming to identify clinically relevant biomarkers for the management of the disease.

## Competing interests

The authors declare that they have no competing interests.

## Authors’ contributions

RM and FJC designed the study, performed DNA methylation analyses, coordinated tissue sampling and wrote the manuscript. MV and SR carried out pyrosequencing experiments. AG carried out bioinformatic analyses. VR and ME participated in the design of the study and helped drafting the manuscript. MK and GS coordinated tissue sampling and analyzed clinical data. WP and AB performed the neuropathological characterization of the samples and the IHC of the tissues. All authors read and approved the manuscript as submitted.

## Pre-publication history

The pre-publication history for this paper can be accessed here:

http://www.biomedcentral.com/1471-2407/14/213/prepub

## Supplementary Material

Additional file 1: Table S1 Pyrosequencing primers used for the validation of selected candidates. Click here for file

Additional file 2: Figure S1 BRAF V600E positivity observed in patient PXA-3 (left image); and CD34 positivity detected in patient PXA-1 (right image). Click here for file

Additional file 3: Figure S2 Bisulphite genomic sequencing of *MGMT* promoter sequence in the precursor (PXA-4) and anaplastic (PXA-5) samples. Squares represent CpG sites along the promoter sequence, displaying methylated (black) or unmethylated (white) status. Click here for file

Additional file 4: Table S2 List of hypermethylated genes in the anaplastic PXA cases. Click here for file

Additional file 5: Table S3 List of hypermethylated genes in benign PXA cases. Click here for file

## References

[B1] Kepes JJ Rubinstein LJ Eng LF Pleomorphic xanthoastrocytoma: a distinctive meningocerebral glioma of young subjects with relatively favourable prognosis. A study of 12 cases Cancer 1979 44 1839 1852 10.1002/1097-0142(197911)44:5<1839::AID-CNCR2820440543>3.0.CO;2-0 498051

[B2] Giannini C Scheithauer BW Classification and grading of low-grade astrocytic tumors in children Brain Pathol 1977 7 785 798 9161729 10.1111/j.1750-3639.1997.tb01064.x PMC8098338

[B3] Pahapill PA Ramsay DA Del Maestro RF Pleomorphic xanthoastrocytoma: case report and analysis of the literature concerning the efficacy of resection and the significance of necrosis Neurosurgery 1996 38 822 828 10.1227/00006123-199604000-00038 8692406

[B4] Koeller KK Henry J From the archives of the AFIP: superficial gliomas: radiologic-pathologic correlation. Armed Forces Institute of Pathology Radiographics 2001 21 1533 1556 10.1148/radiographics.21.6.g01nv051533 11706224

[B5] Gil-Gouveia R Cristino N Farias JP Trindade A Ruivo NS Pimentel J Pleomorphic xanthoastrocytoma of the cerebellum: illustrated review Acta Neurochir (Wien) 2004 146 1241 1244 10.1007/s00701-004-0366-5 15455217

[B6] Herpers MJ Freling G Beuls EA Pleomorphic xanthoastrocytoma in the spinal cord. Case report J Neurosurg 2004 80 564 569 8113873 10.3171/jns.1994.80.3.0564

[B7] Yeh DJ Hessler RB Stevens EA Lee MR Composite pleomorphic xanthoastrocytoma-ganglioglioma presenting as a suprasellar mass: case report Neurosurgery 2003 52 1465 1468 10.1227/01.NEU.0000065138.24985.53 12762893

[B8] Giannini C Scheithauer BW Burger PC Brat DJ Wollan PC Lach B O’Neill BP Pleomorphic xanthoastrocytoma: what do we really know about it? Cancer 1999 85 2033 2045 10.1002/(SICI)1097-0142(19990501)85:9<2033::AID-CNCR22>3.3.CO;2-Q 10223246

[B9] Esteller M Fraga MF Paz MF Campo E Colomer D Novo FJ Calasanz MJ Galm O Guo M Benitez J Herman JG Cancer epigenetics and methylation Science 2002 297 1807 1808 12229925 10.1126/science.297.5588.1807d

[B10] Jones PA Baylin SB The fundamental role of epigenetic events in cancer Nat Rev Genet 2002 3 415 487 12042769 10.1038/nrg816

[B11] Esteller M Epigenetics provides a new generation of oncogenes and tumour-suppressor genes Br J Cancer 2007 96 Suppl R26 R30 17393582

[B12] Jones PA Baylin SB The epigenomics of cancer Cell 2007 128 683 692 10.1016/j.cell.2007.01.029 17320506 PMC3894624

[B13] Cadieux B Ching TT Van den Berg SR Costello JF Genome-wide hypomethylation in human glioblastomas associated with specific copy number alteration, methylenetetrahydrofolate reductase allele status, and increased proliferation Cancer Res 2006 66 8469 8476 10.1158/0008-5472.CAN-06-1547 16951158

[B14] Esteller M Corn PG Baylin SB Herman JG A gene hypermethylation profile of human cancer Cancer Res 2001 61 3225 3229 11309270

[B15] Waha A Güntner S Huang TH Yan PS Arslan B Pietsch T Wiestler OD Waha A Epigenetic silencing of the protocadherin family member PCDH-gamma-A11 in astrocytomas Neoplasia 2005 7 193 199 10.1593/neo.04490 15799819 PMC1501138

[B16] Horiguchi K Tomizawa Y Tosaka M Ishiuchi S Kurihara H Mori M Saito N Epigenetic inactivation of RASSF1A candidate tumor suppressor gene at 3p21.3 in brain tumors Oncogene 2003 22 7862 7865 10.1038/sj.onc.1207082 14586413

[B17] Stone AR Bobo W Brat DJ Devi NS Van Meir EG Vertino PM Aberrant methylation and down-regulation of TMS1/ASC in human glioblastoma Am J Pathol 2004 165 1151 1161 10.1016/S0002-9440(10)63376-7 15466382 PMC1618625

[B18] Nakamura M Sakaki T Hashimoto H Nakase H Ishida E Shimada K Konishi N Frequent alterations of the p14(ARF) and p16(INK4a) genes in primary central nervous system lymphomas Cancer Res 2001 61 6335 6339 11522621

[B19] Alaminos M Dávalos V Ropero S Setién F Paz MF Herranz M Fraga MF Mora J Cheung NK Gerald WL Esteller M EMP3, a myelin-related gene located in the critical 19q13.3 region, is epigenetically silenced and exhibits features of a candidate tumor suppressor in glioma and neuroblastoma Cancer Res 2005 65 2565 2571 10.1158/0008-5472.CAN-04-4283 15805250

[B20] Tews B Roerig P Hartmann C Hahn M Felsberg J Blaschke B Sabel M Kunitz A Toedt G Neben K Benner A von Deimling A Reifenberger G Lichter P Hypermethylation and transcriptional downregulation of the CITED4 gene at 1p34.2 in oligodendroglial tumours with allelic losses on 1p and 19q Oncogene 2007 26 5010 5016 10.1038/sj.onc.1210297 17311001

[B21] Alonso ME Bello MJ Gonzalez-Gomez P Arjona D Lomas J de Campos JM Isla A Sarasa JL Rey JA Aberrant promoter methylation of multiple genes in oligodendrogliomas and ependymomas Cancer Genet Cytogenet 2003 144 134 142 10.1016/S0165-4608(02)00928-7 12850376

[B22] Simpson DJ Clayton RN Farrell WE Preferential loss of death associated Protein kinase expression in invasive pituitary tumours is associated with either CpG island methylation or homozygous deletion Oncogene 2002 21 1217 1224 10.1038/sj.onc.1205195 11850841

[B23] Lindemann C Hackmann O Delic S Schmidt N Reifenberger G Riemenschneider MJ SOCS3 promoter methylation is mutually exclusive to EGFR amplification in gliomas and promotes glioma cell invasion through STAT3 and FAK activation Acta Neuropathol 2011 122 241 251 10.1007/s00401-011-0832-0 21590492

[B24] Laffaire J Everhard S Idbaih A Criniere E Marie Y de Reynies A Schiappa R Mokhtari K Hoang-Xuan K Sanson M Delattre JY Thillet J Ducray F Methylation profiling identifies 2 groups of gliomas according to their tumorigenesis Neuro Oncol 2011 13 8498 10.1093/neuonc/noq110 PMC3018904 20926426

[B25] Esteller M Hamilton SR Burger PC Baylin SB Herman JG Inactivation of the DNA repair gene O6-methylguanine-DNA methyltransferase by promoter hypermethylation is a common event in primary human neoplasia Cancer Res 1999 59 793 797 10029064

[B26] Martinez R Schackert G Yaya-Tur R Rojas-Marcos I Herman JG Esteller M Frequent hypermethylation of the DNA repair gene MGMT in long-term survivors of glioblastoma multiforme J Neurooncol 2006 83 91 93 17164975 10.1007/s11060-006-9292-0

[B27] Uhlmann K Rohde K Zeller C Szymas J Vogel S Marczinek K Thiel G Nürnberg P Laird PW Distinct methylation profiles of glioma subtypes Int J Cancer 2003 106 52 59 10.1002/ijc.11175 12794756

[B28] Marucci G Morandi L Assessment of MGMT promoter methylation status in pleomorphic xanthoastrocytoma J Neurooncol 2011 105 397 400 10.1007/s11060-011-0605-6 21626073

[B29] Martinez R Martin-Subero JI Rohde V Kirsch M Alaminos M Fernandez AF Ropero S Schackert G Esteller M A microarray-based DNA methylation study of glioblastoma multiforme Epigenetics 2009 4 255 264 19550145 10.4161/epi.9130

[B30] Carmona FJ Villanueva A Vidal A Muñoz C Puertas S Penin RM Gomà M Lujambio A Piulats JM Mesía R Sánchez-Céspedes M Manós M Condom E Eccles SA Esteller M Epigenetic disruption of cadherin-11 in human cancer metastasis J Pathol 2012 228 230 240 10.1002/path.4011 22374749 PMC3467766

[B31] Rodríguez-Mena R Joanes-Alepuz V Barbella-Aponte R Pérez-Valles A Pleomorphic xanthoastrocytoma with intraventricular extension and anaplastic transformation in an adult patient: Case report Neurocirugia (Astur) 2012 23 203 210 10.1016/j.neucir.2011.08.003 22867919

[B32] Ng WH Lim T Yeo TT Pleomorphic xanthoastrocytoma in elderly patients may portend a poor prognosis J Clin Neurosci 2008 15 476 478 10.1016/j.jocn.2006.09.012 18255294

[B33] Kleihues P Louis DN Scheithauer BW Rorke LB Reifenberger G Burger PC Cavenee WK The WHO classification of tumors of the nervous system J Neuropathol Exp Neurol 2002 61 215 225 11895036 10.1093/jnen/61.3.215

[B34] Ohgaki H Schäuble B Zur Hausen A Von Ammon K Kleihues P Genetic alterations associated with the evolution and progression of astrocytic brain tumours Virchows Arch 1995 427 113 118 7582239 10.1007/BF00196514

[B35] Skiriute D Vaitkiene P Saferis V Asmoniene V Skauminas K Deltuva VP Tamasauskas A MGMT, GATA6, CD81, DR4, and CASP8 gene promoter methylation in glioblastoma BMC Cancer 2012 12 218 10.1186/1471-2407-12-218 22672670 PMC3404983

[B36] Skiriutė D Vaitkienė P Ašmonienė V Steponaitis G Deltuva VP Tamašauskas A Promoter methylation of AREG, HOXA11, hMLH1, NDRG2, NPTX2 and Tes genes in glioblastoma J Neurooncol 2013 113 441 449 10.1007/s11060-013-1133-3 23624749

[B37] Mueller W Nutt CL Ehrich M Riemenschneider MJ von Deimling A van den Boom D Louis DN Downregulation of RUNX3 and TES by hypermethylation in glioblastoma Oncogene 2007 26 583 593 10.1038/sj.onc.1209805 16909125

[B38] Qiu H Zhu J Yuan C Yan S Yang Q Kong B Frequent hypermethylation and loss of heterozygosity of the testis derived transcript gene in ovarian cancer Cancer Sci 2010 101 1255 1260 10.1111/j.1349-7006.2010.01497.x 20180808 PMC11159749

[B39] Weeks RJ Kees UR Song S Morison IM Silencing of TESTIN by dense biallelic promoter methylation is the most common molecular event in childhood acute lymphoblastic leukaemia Mol Cancer 2010 9 163 10.1186/1476-4598-9-163 20573277 PMC3224738

[B40] de Sousa E Melo F Colak S Buikhuisen J Koster J Cameron K de Jong JH Tuynman JB Prasetyanti PR Fessler E van den Bergh SP Rodermond H Dekker E van der Loos CM Pals ST van de Vijver MJ Versteeg R Richel DJ Vermeulen L Medema JP Methylation of cancer-stem-cell-associated Wnt target genes predicts poor prognosis in colorectal cancer patients Cell Stem Cell 2011 9 476 485 10.1016/j.stem.2011.10.008 22056143

[B41] Küry P Greiner-Petter R Cornely C Jürgens T Müller HW Mammalian achaete scute homolog 2 is expressed in the adult sciatic nerve and regulates the expression of Krox24, Mob-1, CXCR4, and p57kip2 in Schwann cells J Neurosci 2012 22 7586 7595 12196582 10.1523/JNEUROSCI.22-17-07586.2002 PMC6758000

[B42] Hegi ME Diserens AC Godard S Dietrich PY Regli L Ostermann S Otten P Van Melle G de Tribolet N Stupp R Clinical trial substantiates the predictive value of O-6-methylguanine-DNA methyltransferase promoter methylation in glioblastoma patients treated with temozolomide Clin Cancer Res 2004 10 1871 1874 10.1158/1078-0432.CCR-03-0384 15041700

[B43] Sallinen SL Sallinen P Haapasalo H Kononen J Karhu R Helén P Isola J Accumulation of genetic changes is associated with poor prognosis in grade II astrocytomas Am J Pathol 1997 151 1799 1807 9403731 PMC1858343

[B44] Rey JA Bello MJ de Campos JM Kusak ME Moreno S Chromosomal composition of a series of 22 human low-grade gliomas Cancer Genet Cytogenet 1987 29 223 237 10.1016/0165-4608(87)90233-0 3677044

[B45] Yin XL Hui AB Liong EC Ding M Chang AR Ng HK Genetic imbalances in pleomorphic xanthoastrocytoma detected by comparative genomic hybridization and literature review Cancer Genet Cytogenet 2002 132 14 19 10.1016/S0165-4608(01)00512-X 11801302

[B46] Weber RG Hoischen A Ehrler M Zipper P Kaulich K Blaschke B Becker AJ Weber-Mangal S Jauch A Radlwimmer B Schramm J Wiestler OD Lichter P Reifenberger G Frequent loss of chromosome 9, homozygous CDKN2A/p14(ARF)/CDKN2B deletion and low TSC1 mRNA expression in pleomorphic xanthoastrocytomas Oncogene 2007 26 1088 1097 10.1038/sj.onc.1209851 16909113

[B47] Grau E Balaguer J Canete A Martinez F Orellana C Oltra S Hernandez M Castel V Subtelomeric analysis of pediatric astrocytoma: subchromosomal instability is a distinctive feature of pleomorphic xanthoastrocytoma J Neurooncol 2007 93 175 182 19099200 10.1007/s11060-008-9763-6

[B48] Schindler G Capper D Meyer J Janzarik W Omran H Herold-Mende C Schmieder K Wesseling P Mawrin C Hasselblatt M Louis DN Korshunov A Pfister S Hartmann C Paulus W Reifenberger G von Deimling A Analysis of BRAF V600E mutation in 1,320 nervous system tumors reveals high mutation frequencies in pleomorphic xanthoastrocytoma, ganglioglioma and extra-cerebellar pilocytic astrocytoma Acta Neuropathol 2011 121 397 405 10.1007/s00401-011-0802-6 21274720

[B49] Rodríguez-Paredes M Esteller M Cancer epigenetics reaches mainstream oncology Nat Med 2010 17 330 339 21386836 10.1038/nm.2305

[B50] Carmona FJ Esteller M DNA methylation in early neoplasia Cancer Biomark 2011 9 101 111 22112471 10.3233/CBM-2011-0184 PMC13015994

[B51] Martinez R Setien F Voelter C Casado S Quesada MP Schackert G Esteller M CpG island promoter hypermethylation of the pro-apoptotic gene caspase-8 is a common hallmark of relapsed glioblastoma multiforme Carcinogenesis 2007 28 1264 1268 10.1093/carcin/bgm014 17272309

