# Supplementary figures and images for: DNA methylation alterations in grade II- and anaplastic pleomorphic xanthoastrocytoma
Source: BMC Cancer. 2014 Mar 20;14:213. doi: 10.1186/1471-2407-14-213 (PMC4000050; doi:10.1186/1471-2407-14-213)

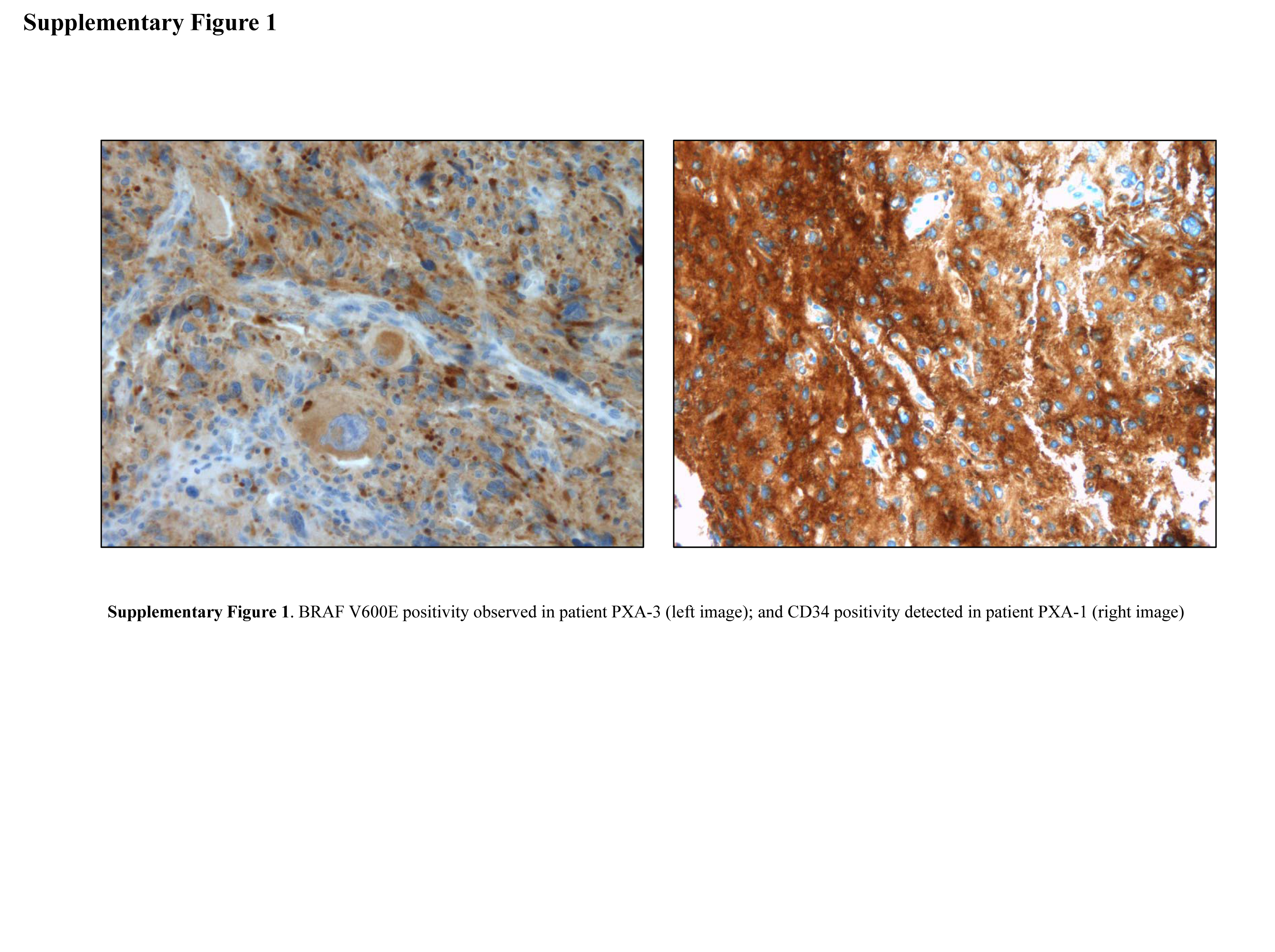

Supplement: Additional file 2: Figure S1 — BRAF V600E positivity observed in patient PXA-3 (left image); and CD34 positivity detected in patient PXA-1 (right image). [file 1471-2407-14-213-S2.tiff]

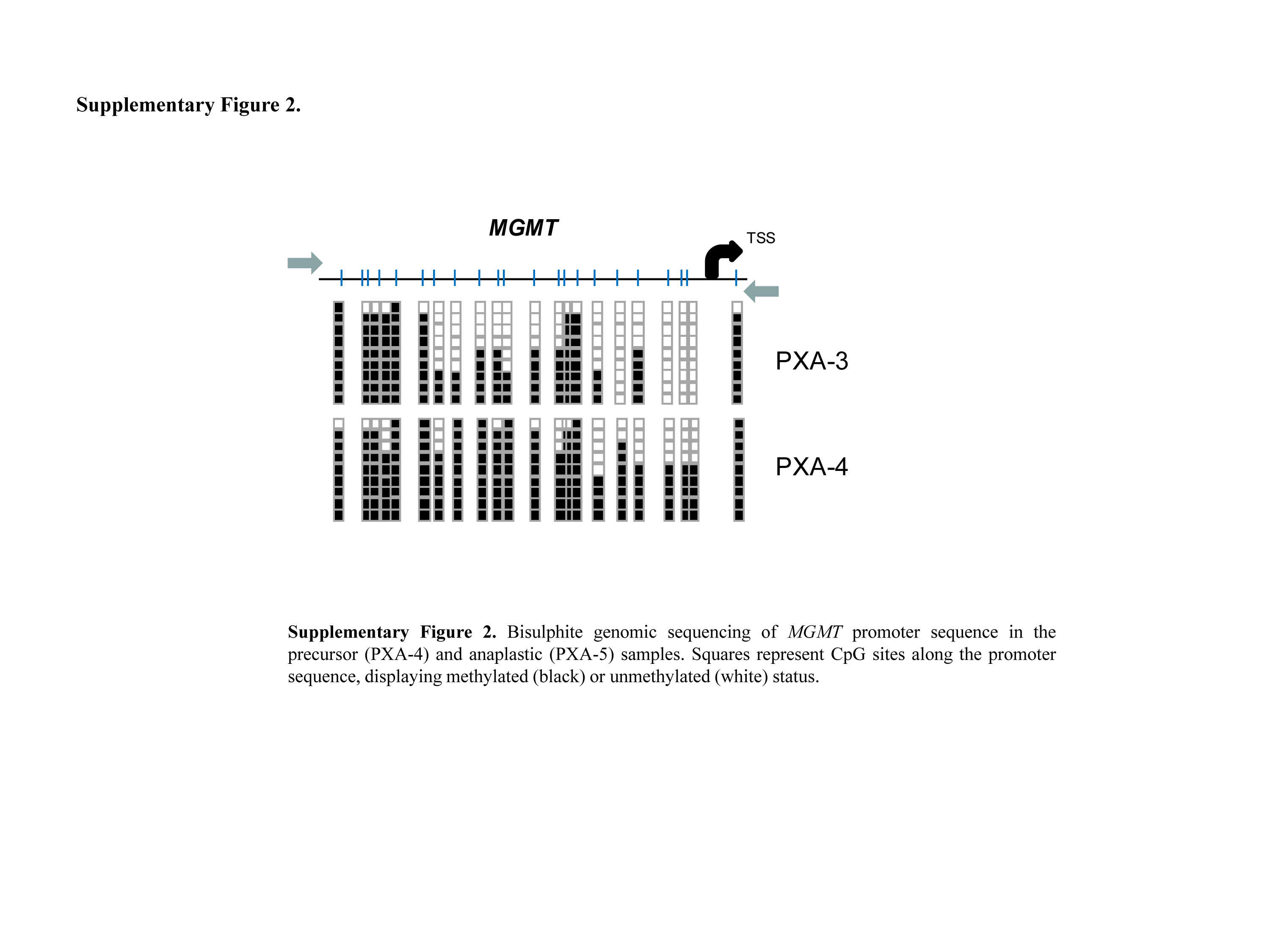

Supplement: Additional file 3: Figure S2 — Bisulphite genomic sequencing of MGMT promoter sequence in the precursor (PXA-4) and anaplastic (PXA-5) samples. Squares represent CpG sites along the promoter sequence, displaying methylated (black) or unmethylated (white) status. [file 1471-2407-14-213-S3.tiff]
